# multiGSEA: a GSEA-based pathway enrichment analysis for multi-omics data

**DOI:** 10.1186/s12859-020-03910-x

**Published:** 2020-12-07

**Authors:** Sebastian Canzler, Jörg Hackermüller

**Affiliations:** Department of Molecular Systems Biology, Helmholtz-Centre for Environmental Research - UFZ, Permoserstraße 15, 04318 Leipzig, Germany

**Keywords:** Pathway enrichment, GSEA, Multi-omics, Bioconductor, Software, R

## Abstract

**Background:**

Gaining biological insights into molecular responses to treatments or diseases from omics data can be accomplished by gene set or pathway enrichment methods. A plethora of different tools and algorithms have been developed so far. Among those, the gene set enrichment analysis (GSEA) proved to control both type I and II errors well. In recent years the call for a combined analysis of multiple omics layers became prominent, giving rise to a few multi-omics enrichment tools. Each of these has its own drawbacks and restrictions regarding its universal application.

**Results:**

Here, we present the multiGSEA package aiding to calculate a combined GSEA-based pathway enrichment on multiple omics layers. The package queries 8 different pathway databases and relies on the robust GSEA algorithm for a single-omics enrichment analysis. In a final step, those scores will be combined to create a robust composite multi-omics pathway enrichment measure. multiGSEA supports 11 different organisms and includes a comprehensive mapping of transcripts, proteins, and metabolite IDs.

**Conclusions:**

With multiGSEA we introduce a highly versatile tool for multi-omics pathway integration that minimizes previous restrictions in terms of omics layer selection, pathway database availability, organism selection and the mapping of omics feature identifiers. multiGSEA is publicly available under the GPL-3 license at https://github.com/yigbt/multiGSEA and at bioconductor: https://bioconductor.org/packages/multiGSEA.

## Background

When measuring molecular responses to a certain treatment or gaining insights into clinical phenotypes, gene set or pathway enrichment techniques are tools of first choice to infer mechanistic biological information from high-dimensional molecular omics data. Through different statistical techniques, such as over-representation analysis (ORA) or gene set enrichment analysis (GSEA), these methods are capable of identifying specific sets of genes or molecular response/signaling pathways that are triggered upon a certain treatment or disease. These sets might represent specific molecular functions, as defined by Gene Ontology (GO) [1], biological processes or experimentally derived gene sets which are publicly available in databases such as Reactome [2] or the Molecular Signature Database (MSigDB) [3].

Up to date nearly one hundred algorithms have been developed for gene set or pathway enrichment, each of which has its own strengths and weaknesses. In principle, these methods can be grouped into two distinct classes: (1) pure gene set enrichment, where the algorithms solely focus on a plain list of features and (2) topology-based enrichment, where algorithms include additional information derived from pathway or network databases, e.g., which genes or proteins are directly connected and how they influence each other. There are several comprehensive reviews on this topic available, see for example [4, 5]. Besides a plain quality assessment of different enrichment techniques, this review also evaluated the robustness of available methods, i.e., how error-prone these methods are w.r.t. the prediction of either false positive or false negative gene sets or pathways. The popular GSEA method showed a decent quality in terms of rank- and *p* value-based pathway enrichment but moreover was the only method not found to produce any false prediction [5].

Over the last decade, combined analysis of molecular responses through the integration of multiple omics types has become prevalent, e.g. combining transcriptomics, proteomics, and metabolomics. This is becoming necessary since single-omics analyses will only measure biomolecules of a specific type and will often not even detect its entirety but only a subset thereof. Furthermore, the response time and the life-span of biomolecules varies substantially within and between single omics layers. Thus, only the combined analysis of several molecular layers through multi-omics measurements reliably allows to uncover a significant fraction of cellular effects [6].

There are a few integration tools available that incorporate pathway knowledge to interpret multi-omics datasets like *PaintOmics* [7] or *IMPaLA* [8]. These methods exhibit several limitations hampering their unrestricted application. *PaintOmics*, for example, is capable of including several different omics layers into its pathway enrichment analysis, but solely relies on pathway definitions from the KEGG database. Furthermore, impacted pathways are determined based on Fisher’s exact test, which was shown to be particularly prone to reporting false positive pathway enrichments [5]. *IMPaLA* on the contrary supports the analysis of a range of different pathway databases but is limited to two different omics input layers, allowing to integrate either transcriptome and metabolome or proteome and metabolome. While *PaintOmics* is applicable to several model organisms (mouse, rat, fruit fly, etc.) *IMPaLA* is restricted to human pathway definitions only.

Here we introduce the multiGSEA R package that provides multi-omics-based pathway enrichment employing the robust GSEA algorithm and allowing to use pathway or gene set definitions from several curated databases. In its current version multiGSEA is applicable to a combination of transcriptome, proteome, and metabolome data measured in 11 different organisms, including human, mouse, or rat.

## Implementation and workflow

**Fig. 1:**
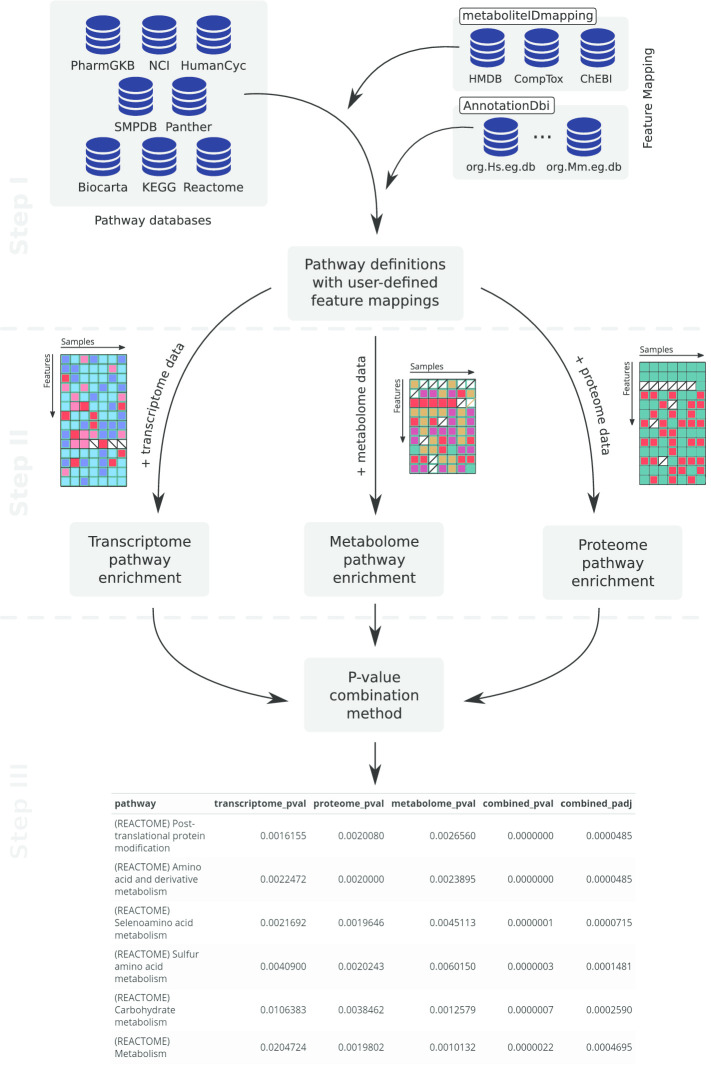
Figure illustrating the workflow of the multiGSEA package. In step 1 pathway databases are queried, the features are extracted and mapped to user-defined ID formats. Single omics pathway enrichment using a GSEA-based approach is performed in step 2. *p* values from these calculations are combined in step 3 to create a multi-omics pathway enrichment

In principle, the workflow of the multiGSEA package is composed of three essential steps: (1) prepare pathway definitions and omics data (2) single omics gene set enrichment analysis (3) combined multi-omics enrichment. These steps are graphically outlined in Fig. 1 and described in detail below:

### Collecting pathway definitions, feature extraction, and mapping

Over the last decades, several pathway databases have been established. Some of which are peer-reviewed and manually curated while others follow a community-based approach to develop and refine pathways. However, often these databases contain their own format in which pathway definitions are provided, making it cumbersome to include multiple databases in an analysis workflow. The ‘graphite‘ ‘R‘ package [9] was designed to bridge this gap since it is able to provide pathway definitions from eight publicly available databases – the numbers of currently available human pathway definitions in these databases are listed in parentheses: KEGG (311) [10], Biocarta (247), Reactome (2208) [2], NCI/Nature Pathway Interaction Database (212) [11], HumanCyc (48682) [12], Panther (94) [13], smpdb (48668) [14], and PharmGKB (66) [15]. Within the first step of the multiGSEA workflow, we make use of the graphite package to retrieve pathway definitions from up to eight public databases.

Depending on the database, pathway features (nodes) are encoded with different ID formats. The KEGG database, for example, uses Entrez Gene IDs for transcripts and proteins while KEGG Compound IDs are used for metabolites. The Reactome database on the contrary stores transcripts and proteins by means of Uniprot identifiers, while ChEBI IDs are used for metabolites. Further metabolite ID formats are CAS numbers and Pubchem IDs. To solve this issue, especially when dealing with multiple pathway databases in a single analysis, we implemented an ID mapping for features of all three supported omics layers. Transcriptomics and proteomics features can be mapped to the following formats: Entrez Gene IDs, Uniprot IDs, Gene Symbols, RefSeq, or Ensembl IDs. The mapping procedure is accomplished by means of the AnnotationDbi Bioconductor package [16] and depends on the loaded annotation database such as org.Hs.eg.db for human [17].

Metabolomic features can be mapped to Comptox Dashboard specific IDs (DTXSID, DTXCID), CAS numbers, Pubchem IDs (CID, SID), KEGG Compound IDs, HMDB IDs, or ChEBI IDs. For enhanced usability we encapsulated this comprehensive metabolite mapping data set in a stand-alone AnnotationHub package called metaboliteIDmapping [18]. In its current version the package contains more than 1.1 million compounds and was collected and integrated from four different databases: Comptox Dashboard^1^,^2^, HMDB^3^, and ChEBI^4^.

### Gene set enrichment analysis

Measured omics data are necessary for the calculation of gene set enrichment scores. These data have to be loaded for each of the omics layers that have been defined in the previous step of extracting pathway-specific features from external databases. Prior to the enrichment score computation, a differential expression analysis has to be performed such that all omics features have an associated fold change and *p* value. The pre-processing step has to be done externally and is not part of the multiGSEA package.

In a second step, multiGSEA calculates the enrichment score by applying the fgsea R package [19] on each omics layer individually. The algorithm *GSEA* in its original form was first described to shed light on the mechanistic basis of Type 2 Diabetes mellitus [20]. The updated and most commonly applied version was introduced by Subramanian et al. [21] two years later. In brief, measured omics features are utilized for a differential expression testing to derive fold changes and associated *p* values. Ranking metrics are then used to calculate the so-called local statistic. In multiGSEA, a ranked feature list is calculated based on the direction of the fold-change and the log-transformed *p* value. Different ranking metrics can be chosen individually and this choice can have strong effects on the outcome of the gene set enrichment analysis [22]. In the following step, GSEA algorithms test whether gene sets accumulate at the top or bottom of those ordered gene vectors. The fgsea version used here is an efficient but yet accurate implementation of the prominent *GSEA* algorithm. Its performance is achieved through implementing a cumulative GSEA-statistic calculation allowing to reuse random gene set samples between different input pathways [19].

After the second part of the multiGSEA workflow, each downloaded pathway has been assigned fgsea-based enrichment scores, *p* values, and adjusted *p* values for each omics layer separately.

### Combined multi-omics enrichment

**Fig. 2:**
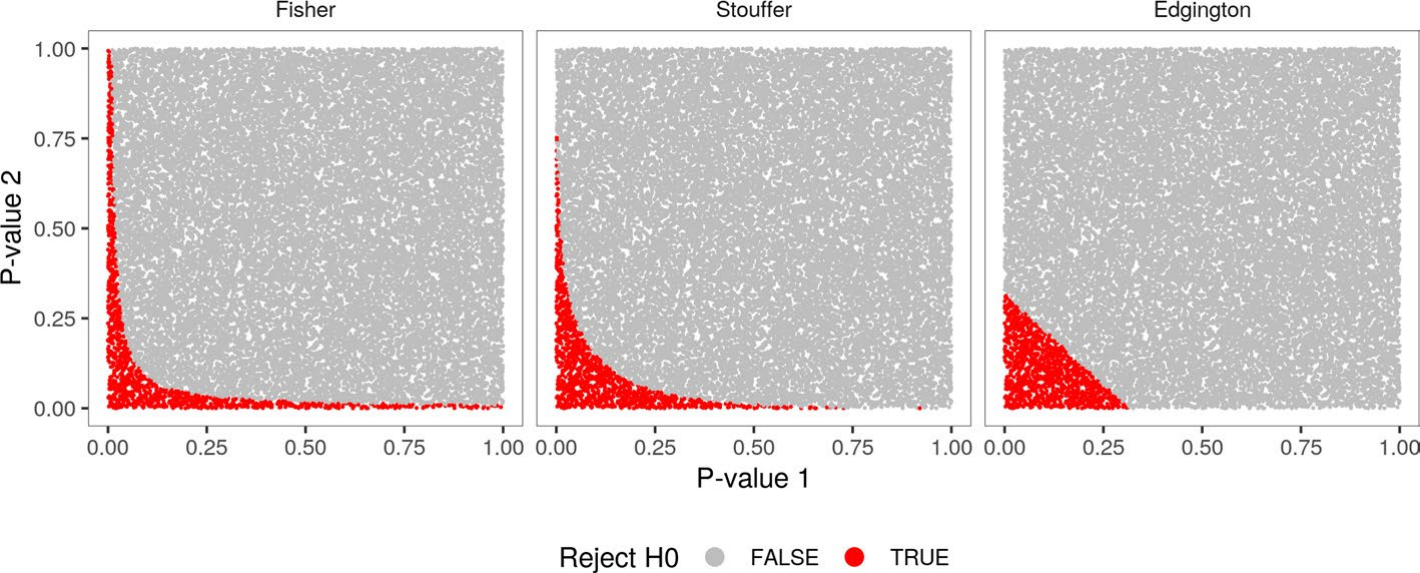
Figure illustrating the difference between Fisher’s combined probability test, Stouffer’s method, and Edgington’s method to aggregate multiple *p* values. Two times 25k *p* values have been randomly chosen to be subsequently combined by either of those three methods. Combined *p* values lower than 0.05 are classified as significant and plotted red

To more comprehensively measure a pathway response, multiGSEA provides different approaches to compute an aggregated *p* value over multiple omics layers. Because no single approach for aggregating *p* values performs best under all circumstances, Loughin proposed basic recommendations on which method to use depending on structure and expectation of the problem [23]. If small *p* values should be emphasized, Fisher’s method should be chosen. In cases where *p* values should be treated equally, Stouffer’s method is preferable. If large *p* values should be emphasized, the user should select Edgington’s method. Figure 2 indicates the difference between those three methods.

A first option is the Fisher’s combined probability test, which uses the *p* values from *k* independent tests (here up to three omics layers) to calculate a test statistic $\mathrm {X}^2_F = -2 \sum _{i=n}^k ln(p_i)$. If all of the null hypotheses of the *k* tests are true, the test statistic will follow a $\mathrm {X}^2$ distribution with $2^k$ degrees of freedom [24]. Fisher’s method is asymmetrically sensitive to small *p* values which results in a bias for aggregated *p* values from multiple studies on the same null hypothesis [25]. This can be seen in Fig. 2 (Fisher) especially in those cases where one of both single *p* value is close to 1 and yet the combined *p* value is still considered to be significant, simply because the other single *p* value is small enough.

To circumvent this asymmetry, multiGSEA can also apply alternative methods: the Z-transform test and the weighted Z-transform test. The first algorithm is also called Stouffer’s method. Both versions make use of the fact that *p* values, ranging from 0 to 1, can be uniquely matched with a value in *Z*, representing a standard normal deviate, and *vice versa*. Each *p* value $p_i$ from *k* independent tests (here omics layer) is converted into deviates $Z_i$, with $Z_i = \Phi ^{-1}(p_i)$ and $\Phi$ being the standard normal cumulative distribution function. Stouffer’s method is defined as:

$$Z_s = \frac{\sum _{i=1}^{k} Z_i}{\sqrt{k}}$$

$Z_s$ follows a standard normal distribution if the null hypothesis is true, and thus can be compared to a standard normal distribution to provide a test of the cumulative evidence [26]. As can be seen in Fig. 2 (Stouffer), this method is smoother and more balanced.

The weighted version of this method is defined by:

$$Z_s = \frac{\sum _{i=1}^{k} w_i * Z_i}{\sqrt{\sum _{i=1}^{k} w_i^2}}$$

It is still an open debate whether the weighted or unweighted version is preferential. However, it has been reported that if the weighted version is used, optimal results are obtained using weights proportional to the square root of the sample sizes [27, 28].

A third alternative method was created by Edgington and relies on untransformed *p* values. It was developed to combine probability values through an additive approach [29]:

$$\frac{S^k}{k!}$$

with

$$S = \sum _{i=1}^{k} p_i$$

and *k* being the number of individual studies. However, this is a rather conservative estimate resulting in combined probability values that are too high when $\sum _{i=1}^k p_i > 1$. To account for this, correction terms were added to the summation:

$$\frac{S^k}{k!} - \left( {\begin{array}{c}k\\ 1\end{array}}\right) \frac{(S-1)^k}{k!} + \left( {\begin{array}{c}k\\ 2\end{array}}\right) \frac{(S-2)^k}{k!} - \dots$$

Plus and minus signs alternate and the series continues until the numerator becomes negative. Finally, the result of this progression is compared to a chosen significance level on whether to reject the null hypothesis or not. As shown in Fig. 2 (Edgington), this combination method is much more conservative compared to Fisher’s and Stouffer’s approach.

A recommendable review on those three (and three additional) aggregation methods was published by Heard and Rubin-Delanchy [30] alongside some practical advises on how to chose a suitable method.

multiGSEA allows to choose from all above described approaches for *p* value combination, which are provided through the metap R package [31]. Both Fisher’s combined probability test and Stouffer’s method have been shown to control both type I and type II errors well upon *p* value combination. However, the weighted Z-transform method was reported superior regarding type II errors [25]. We are not aware of a comparable analysis for Edgington’s method.

After computing combined *p* values, these can be adjusted for multiple testing. Since appropriate methods are available in R base packages, multiGSEA does not provide its own implementation. Type I and type II errors depend on each other and thus reducing type I errors through a *p* value adjustment will likely increase the chance of making a type II error and an appropriate trade-off has to be made [32, 33].

Finally, multiGSEA outputs a plain table listing the pathways with their single-omics and aggregated multi-omics *p* values and adjusted *p* values.

## Results

### Example use case

In the following, we will illustrate a use case scenario on human mitochondrial stress data. A comprehensive vignette of the multiGSEA package can be found in our git repository or at the Bioconductor package website.

Please visit the repository page to report issues, request features or provide other feedback.

#### Installation

For installation we recommend two ways: (i) use the BiocManager package from Bioconductor: 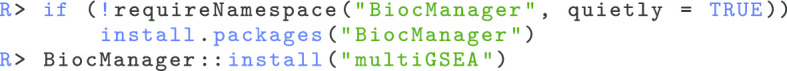


(ii) use the devtools library [34] to install directly from our git repository: 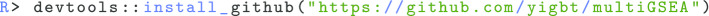


#### Example data and pathway definitions

At the beginning we need to set up several prerequisites. This includes loading the package itself and those packages that are needed to map omics feature IDs such as transcript IDs or metabolite IDs **(i)**. We furthermore need to load the multi-omics data **(ii)** and we have to download the pathway definitions where the enrichment should be calculated on **(iii)**.

**(i)** Load the multiGSEA R package and the packages that are needed for the mapping of omics features IDs: 
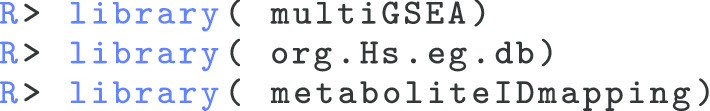


Depending on the organism and omics layer for which an enrichment should be calculated the users might need different packages that provide the necessary ID mapping information. In principle, the mapping of transcript and protein IDs relies on the AnnotationDbi package [16]. In this use case we want to analyse human data, and thus need to load the org.Hs.eb.db package which provides human specific mapping information [17]. When rat or mouse omics data should be analyzed, the user needs to load the corresponding packages org.Rn.eg.db [35] or org.Mm.eg.db [36], respectively. A list of supported organisms and their respective mapping packages can be found in the vignette. The mapping information for metabolites is independent of the analyzed organism and only needs to be loaded when metabolomics data is used in the multi-omics enrichment. The metabolite mapping information is provided by the metaboliteIDmapping R package [18].

**(ii)** Load the multi-omics data sets: 
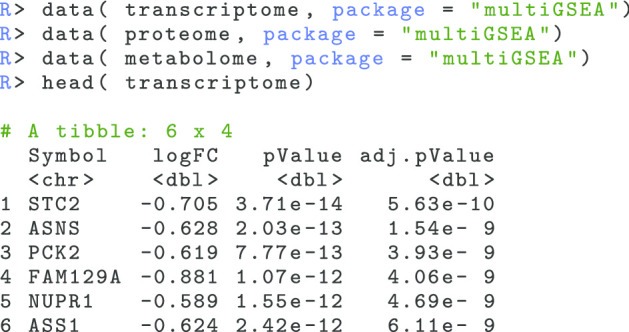


The multi-omics data set that is used in this example was originally published in its raw form by Quirós et al. [37]. In this publication the authors analyzed the mitochondrial response to four different toxicants, including Actinonin, Diclofenac, FCCP, and Mito-Block (MB), within the transcriptome, proteome, and metabolome layer. The original Actinonin data set was processed and log2 fold changes were calculated for all three omics layers. The processed data sets are deposited within the package and can be accessed with the data() command.

Right after multiple omics data sets are loaded, we need to create a suitable data structure and calculate the ranked omics features for the following GSEA calculation. multiGSEA works with nested lists where each sublist represents an omics layer. Such a data structure is initialized with the initOmicsDataStructure() command: 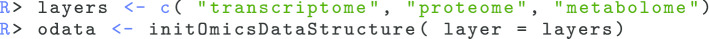


The feature ranks are calculated separately for each of the applied omics layers. In this example we simply use the signed and logarithm transformed *p* value [38] derived from the differentially expression analysis, which is implemented in the rankFeatures() command: 
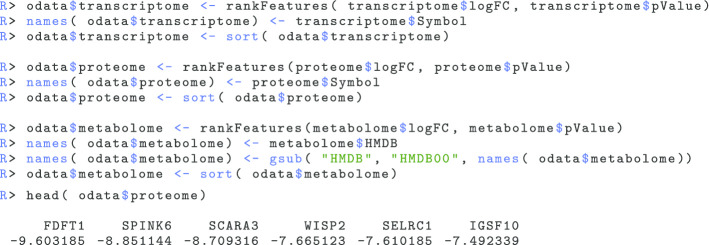


Please note that every other ranking metric is, of course, possible as well, and the choice is up to the user. According to Zyla et al. this decision does also have critical effects on the outcome of the gene set enrichment analysis [22]

**(iii)** Retrieve pathway definitions and map features to the same ID format as in your omics measurements: 
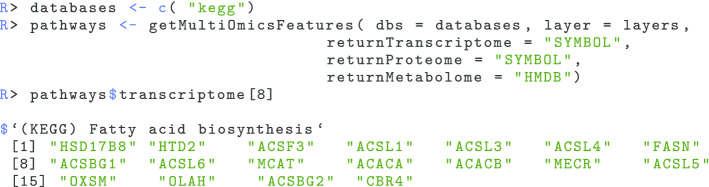


In this use case we merely retrieved KEGG-based pathway definitions but depending on the organism being analyzed up to eight different pathway databases can be queried. The function getMultiOmicsFeatures retrieves the pathway definitions from the specified databases, extracts the omics features thereof, and maps those features to the ID format that is been used in the omics data. Here, it maps Entrez Gene IDs and KEGGCOMP IDs that are used in KEGG pathways for transcripts/proteins and metabolites, respectively, towards Gene Symbols and HMDB identifiers, respectively.

#### Run pathway enrichment

Now that we have ranked omics features and pre-formatted pathway definitions, we can calculate GSEA-based pathway enrichments for each omics layer separately by means of multiGSEA: 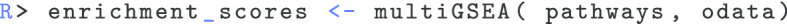


The pathway enrichment within multiGSEA is done by the fgsea package [19]. This package allows to efficiently and accurately calculate arbitrarily low GSEA *p* values for a collection of feature sets. This speedup compared to the original GSEA implementation is basically accomplished because generated random gene sets are shared between different input pathways.

The returned data frame enrichment_scores contains nested lists for each analyzed omics layer and each omics-specific sublist contains the complete gene set enrichment analysis for its respective layer.

#### Calculate aggregated *p* values

For further analysis, the function extractPvalues() creates a simple data frame where each row represents a pathway and columns represent omics related *p* values and adjusted *p* values: 
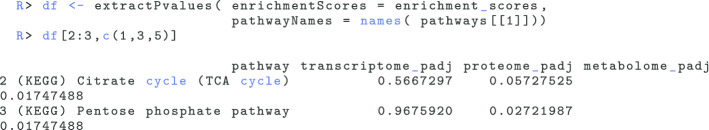


This data structure can then be used to calculate the aggregated *p* values and the adjusted *p* values. As explained in the workflow section covering the combination of multiple omics pathway enrichment *p* values (“Combined multi-omics enrichment” section), multiGSEA utilizes three different *p* value combination methods. By default, combinePvalues() will apply the Z-method or Stouffer’s method [26] which has no bias towards small or large *p* values. The two other options are Fisher’s combined probability test [24] and Edgington’s method [29]. These can be applied by setting the parameter method to “fisher” or “edgington”, respectively. The choice to additionally correct for multiple testing is up to the user. It should be mentioned, however, that *p* value adjustments have an effect on both type I and II errors. Here, we used the p.adjust() command to apply a Benjamini/Hochberg correction [39]. 
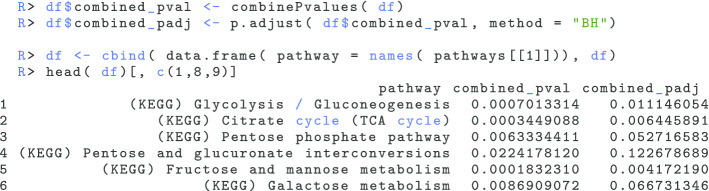


## Conclusions

The presented multiGSEA package substantially helps to minimize the drawbacks and restriction that have been identified in recent multi-omics enrichment tools. It utilizes a robust enrichment algorithm with GSEA that has been shown to keep both the type I and II error rate at a minimum. The multiGSEA package offers, furthermore, a high versatility and flexibility through the accessibility of eight different pathway databases and its support for 11 different model organisms. The user is able to calculate enrichment scores for one, two, or all three provided omics layers and has access to a comprehensive mapping of omics features IDs on the transcriptome, proteome, and metabolite level. Finally, the whole process of data retrieval, mapping of feature IDs, the calculation of pathway enrichments, and the combination of those enrichment scores is wrapped into an intuitive and easy-to-use workflow which considerably simplifies the inference of biological meaning from multi-omics data sets.

## Availability and requirements

The multiGSEA package is entirely written in R and available under the GPL-3 license. The package is part of the Bioconductor project to provide open source software for bioinformatics at https://bioconductor.org/packages/multiGSEA. The current development version of the package can be found on our GitHub page at https://github.com/yigbt/multiGSEA or in the Bioconductor development branch at https://bioconductor.org/packages/devel/bioc/html/multiGSEA.html.

**Project name:** multiGSEA

**Project home page:** https://github.com/yigbt/multiGSEA,

https://bioconductor.org/packages/multiGSEA

**Operating system(s):** Platform independent

**Programming language:** R

**Other requirements:** No

**License:** GNU GPL V3.

**Any restrictions to use by non-academics:** No

## Data Availability

The datasets that have been analyzed in the use case and the vignette were originally published in its raw form by Quirós et al. in 2017: https://doi.org/10.1083/jcb.201702058. Transcriptomics data was downloaded from NCBI Geo: https://www.ncbi.nlm.nih.gov/geo/query/acc.cgi?acc=GSE84631. Proteomics data was retrieved from ProteomeXchange Consortium: http://proteomecentral.proteomexchange.org/cgi/GetDataset?ID=PXD006293. The metabolomics data was retrieved from the supplementary material.

## Abbreviations

ChEBI: Chemical entities of biological interest
GSEA: Gene set enrichment analysis
GO: Gene ontology
HMDB: Human metabolome database
KEGG: Kyoto encyclopedia of genes and genomes
MSigDB: Molecular signature database
ORA: Over-representation analysis

## Footnotes

1 ftp://newftp.epa.gov/COMPTOX/Sustainable_Chemistry_Data/Chemistry_Dashboard/PubChem_DTXSID_mapping_file.txt.

2 ftp://newftp.epa.gov/COMPTOX/Sustainable_Chemistry_Data/Chemistry_Dashboard/2019/April/DSSToxSDF.zip.

3 http://www.hmdb.ca/system/downloads/current/hmdb_metabolites.zip.

4 ftp://ftp.ebi.ac.uk/pub/databases/chebi/Flat_file_tab_delimited/database_accession.tsv.
